# Improving Discrimination in Predicting Level of Care Needed for Patients Admitted with Pneumonia

**DOI:** 10.1007/s11606-025-09610-7

**Published:** 2025-05-22

**Authors:** David E. Katz, Gideon Leibner, Nechama Kaufman, Yaakov Esayag, Shuli Brammli-Greenberg, Adam J. Rose

**Affiliations:** 1 Department of Internal Medicine, Shaare Zedek Medical Center and Faculty of Medicine, Hebrew University of Jerusalem, Jerusalem, Israel; 2 Department of Surgery, Hadassah University Medical Center, Jerusalem, Israel; 3 Department of Quality and Patient Safety, Shaare Zedek Medical Center, Jerusalem, Israel; 4 Department of Emergency Medicine, Shaare Zedek Medical Center, Jerusalem, Israel; 5 Meuhedet Health Care Services HMO, Jerusalem, Israel; 6 School of Public Health, Faculty of Medicine, Hebrew University of Jerusalem, Jerusalem, Israel; 7 Department of Internal Medicine, Shaare Zedek Medical Center, Jerusalem, Israel

**Keywords:** risk adjustment, pneumonia, internal medicine

## Abstract

**Background:**

Various risk stratification scores are used to predict outcomes among patients with pneumonia. We have developed a novel model that predicts the risk of death or intensive care unit transfer in internal medicine.

**Objective:**

To compare the ability of two prediction models to predict clinical outcomes in patients admitted for pneumonia, using information available at the time of admission.

**Design:**

Comparison of two prediction models.

**Participants:**

3856 pneumonia admissions to the internal medicine service of a tertiary medical center.

**Main Measures:**

We compared the ability of two scores to predict in-hospital mortality and escalation of care (e.g., to the intensive care unit) among patients admitted for pneumonia. One was the CURB-65 score, which is currently in use at our hospital. The other score was one we developed, based on the Elixhauser case mix adjustment model with additional data, such as vital signs and laboratory values.

**Key Results:**

11.8% of patients died in-hospital and 17.7% required an escalation of care. The most common CURB-65 score was 2 (44%), the lowest CURB-65 score ordinarily requiring admission. Our risk prediction score was better than CURB-65 at predicting mortality (c-statistic 0.846 vs. 0.724) and escalation (0.757 vs. 0.633). Our score was able to discriminate among patients classified as similar-risk by the CURB-65 score: of the 1681 patients with a (medium-risk) CURB-65 score of 2, our model placed 180 (11%) into the lowest-risk quintile of patients, and 309 (18%) into the highest-risk quintile.

**Conclusions:**

Our risk stratification tool is calculable with information available in the electronic medical record of most hospitals. The new score was much better able to predict the outcomes of in-hospital mortality and escalation of care among patients admitted for pneumonia, compared to CURB-65.

**Supplementary Information:**

The online version contains supplementary material available at 10.1007/s11606-025-09610-7.

## INTRODUCTION

Pneumonia is one of the most common reasons for hospital admission. In 2018, pneumonia was the fourth most common non-maternity-related reason for hospitalization in the USA.^1^ It has long been a priority to predict which patients with pneumonia are most likely to die or require intensive care. The first major risk stratification tool for pneumonia was the Pneumonia Patient Outcomes Research Team (PORT) score, which is still in use as the Pneumonia Severity Index (PSI). The PSI requires detailed data about the patient, including vital signs, comorbid conditions, and laboratory values.^2^ While it is possible to calculate manually, it takes several minutes to do so. Another score, developed more recently, is the CURB-65 score.^3^ CURB stands for the components of the score, including Confusion, blood Urea nitrogen, Respiratory rate, Blood pressure, and age 65 or older. CURB-65 is easier to calculate than the PSI and performs at a similar level in terms of predicting outcomes such as in-hospital mortality.^4^

Data are now collected in structured form and are available within the hospital computer system—which was not the case when these scores were developed. Thus, it would now be possible to calculate a risk score for each patient soon after arrival in the emergency department and display it to the clinician in real time. In addition, the score could potentially involve a large number of variables—too many to be convenient for manual calculation. We have recently developed a score that is intended to risk stratify across a general population of patients admitted to a large, tertiary-care hospital in Israel.^5^ This score relies on the Elixhauser model,^6^ augmented with additional information such as laboratory values and vital signs. Our model achieves a high level of prediction for in-hospital mortality, escalation of care (e.g., transfer to the intensive care unit), and length of stay in a general population of Internal Medicine (IM) patients.^5^

Here, we examined the use of this score to risk stratify patients with pneumonia. We focused on pneumonia because it represents a relatively large population of patients with a single admission diagnosis, for which risk models already exist. We compared the ability of our score to predict clinical outcomes, including in-hospital mortality and escalation of care, with the CURB-65 score—the score that is currently used in our hospital for this purpose. If it performs better, our new score could easily be displayed to clinicians in real time based on information available in the computer system of any modern hospital. This could be a valuable guide for decisions such as whether to admit the patient, and to what setting.

## STUDY DESIGN AND METHODS

### Database

We have previously described our methods and database.^7^ We used data from the Shaare Zedek Medical Center (SZMC), a large tertiary-care referral hospital in Jerusalem that serves a varied population in terms of ethnicity and socioeconomic status. The present analysis was based on 3856 admissions to the IM service for a diagnosis of pneumonia, which occurred between 01.01.2016–31.12.2019. These dates were chosen to allow us to study care under usual conditions, prior to the influence that COVID-19 had on the Israeli medical system. The study unit was hospitalizations rather than patients, since some patients were admitted more than once.

For the purposes of this study, “internal medicine wards” were defined as the four formal IM departments (A, B, C, and D), geriatrics, cardiology, hematology-oncology, and a short stay unit. The rationale for including these additional units is that some Israeli hospitals do not have such units, and therefore the patients hospitalized at SZMC in these wards would be part of the population served by IM in a different hospital setting. In order to capture the entire spectrum of IM patients, they are included here. This study was approved by the research ethics committee of SZMC (0361–21-SZMC).

### Demographics and Patient Characteristics

Patient deidentified medical information was extracted from the hospital’s electronic medical record. We characterized demographics, dates of hospitalization and discharge, date of birth, gender, and all wards the patient visited during the hospital stay.

### Dependent Variables: Patient Outcomes

There were two dependent variables (outcomes) for this study, each of which was modeled as a separate outcome. Both are binary outcomes. The first outcome was in-hospital mortality. The second was requiring an escalation of care beyond the regular medicine ward. Together, knowing how likely these two outcomes are to occur during a hospitalization gives a strong understanding of a patient’s level of risk, and can therefore be useful to inform clinical management, prognosis, and for risk adjustment purposes.

Our definition of escalation of care was expansive and included any patient who spent part of their hospital stay in the intensive care unit, in the intermediate care unit, who received mechanical ventilation, daytime bi-level positive pressure ventilation (BiPAP), or vasopressors (i.e., medications intended to support blood pressure). Daytime BiPAP was defined as occurring between 8 AM and 8 PM. BiPAP received at night may be needed for disordered breathing during sleep, but BiPAP received during the day is presumably intended as a method to prevent invasive mechanical ventilation. Vasopressors included adrenaline, dobutamine, dopamine, milrinone, noradrenaline, phenylephrine, and vasopressin. Any of these interventions sufficed to show that the patient had at least some degree of critical illness and required intensive intervention. Our group has previously published about the relationship between bed location and the receipt of mechanical ventilation, daytime BiPAP, and vasopressors.^8^ Some patients experienced an escalation of care and then died during the same hospitalization. Such patients were included in both analyses.

### Independent Variable: Enhanced Elixhauser Index

We have previously described our adaptation of the Elixhauser Index to use for case mix adjustment in the IM service of SZMC.^7^ In a subsequent article, we described our development and validation of an enhanced model, which started with the Elixhauser index but added information such as vital signs and laboratory values at the time of admission.^5^ These variables were chosen because they are almost universally available for patients admitted to IM.

Briefly, the original Elixhauser index is based on 29 comorbid conditions, which are identified using ICD-9 codes.^6^ Our modification of this model greatly improved its ability to predict the outcomes we are examining in the present manuscript, compared to the original Elixhauser model.^5^

### Independent Variable: CURB-65 Score

The CURB-65 score is one of the most commonly used risk stratification tools for patients admitted with community-acquired pneumonia.^3^ Based on the British Thoracic Society assessment tool, a six-point “CURB-65” severity score (**C**onfusion, **U**rea > 7 mmol/l, **R**espiratory rate ≥ 30/min, low **B**lood pressure (diastolic blood pressure ≤ 60 mmHg or systolic blood pressure < 90 mmHg), and **A**ge ≥ 65 years was calculated, and one point was given for each feature present (range 0–5 points).^3^ This score has been used to stratify patients into distinct risk groups. The CURB-65 score has been validated over the years for the management of pneumonia.^4^ Simplified scores based on the CURB-65, such as the CRB-65 and the A-DROP score, have also been used for severity adjustment^9^ and for prediction of mortality in hospitalized patients with pneumonia.^10^ The CURB-65 score has even been combined with the procalcitonin and albumin test to assess short-term mortality in hospitalized elderly patients with infectious disease.^11^ However, due to its simplicity and supportive studies, the CURB-65 score is still widely used and is the one deployed for use at SZMC. For our analysis, confusion was obtained from the “mental condition” section of the Norton score; a “1” was assigned to the patient if they were categorized as stuporous or confused, or else they were assigned “0.”

### Statistical Analyses

We began by examining the frequency of basic information (e.g., sex, age) and the study outcomes among the 3856 hospitalizations for pneumonia included in our database. For each of these hospitalizations, we calculated a predicted risk of in-hospital mortality and of escalation of care using the case mix model we had previously developed.^5^ We also assigned a CURB-65 score to each of these hospitalizations.

We divided patients into quintiles based on our case mix model and compared these quintiles of risk with the CURB-65 scores—both for in-hospital mortality and for escalation of care. We plotted receiver operating characteristic (ROC) curves for our case mix model and for the CURB-65 score to predict the study outcomes in this population and reported the c-statistic, a measure of model fit for a logistic model. We also characterized model calibration, which can be thought of as a measure of whether the model systematically overestimates risk in lower-risk or in higher-risk groups, and by how much, using a Hosmer–Lemeshow test.^12^ Analyses were performed using SPSS version 24 and R Studio version 1.3.1093.

## RESULTS

Characteristics of the 3856 hospitalizations for pneumonia during the study period can be seen in Table 1. Most of the patients were elderly, with a mean age of 80 years. The sex distribution was balanced. The most common CURB-65 score was 2 (44%), which is the lowest CURB-65 score routinely recommended for hospital admission. Interestingly, 34.5% of the patients had a CURB-65 score of less than 2, implying that at least some of them may not have required hospital admission for purely medical reasons.

**Table 1:** Population Characteristics of 3856 Adult Patients Hospitalized for Pneumonia. Number and Percent Given Except Where Noted

Characteristic	
Age at hospitalization (mean, SD)	81 (17.81)
Sex	
Female	1885 (49%)
Male	1971 (51%)
Died in hospital	455 (11.8%)
Escalation of care	682 (17.7%)
Length of stay, days (mean, SD)	8.15 (10.48)
Length of stay, days (median, IQR)	5.10 (3.00, 9.00)
CURB-65 score	
0	329 (8.5%)
1	1021 (26%)
2	1681 (44%)
3	702 (18%)
4	114 (3.0%)
5	9 (0.2%)

The risk of death compared to the risk of escalation of care for our study population using the expanded Elixhauser model can be seen in Table 2. This table could be used to identify appropriate patients for treatment as outpatients or in a hospital-in-the-home setting, using our case mix score. For example, taking the top left 2 × 2 cells identifies close to 28.7% (1106/3856) of the lower-risk patients who could potentially be sent home from the ED to receive treatment there. By definition, all 1106 of these patients have a predicted risk of in-hospital mortality of less than 3.7% and a risk of escalation of care of less than 10.6%. A different set of cells (e.g., the top 2 rows and 3 leftmost columns) could be used to select a different group of patients, based on one’s risk tolerance and how sick a group of patients one’s home treatment service is equipped to handle.

**Table 2:** Calculated Risk of Death Compared with Risk of Escalation of Care, Based on the Risk Adjustment Model

		Risk of escalation of care					
		Quintile 1 Lowest risk (1.9–6.5%)	Quintile 2 (6.5–10.6%)	Quintile 3 (10.6–15.8%)	Quintile 4 (15.8–26.7%)	Quintile 5 Highest risk (26.7–96.8%)	Total
Risk of in-hospital mortality	Quintile 1 Lowest risk (0.3–1.4%)	480	204	82	24	6	796
	Quintile 2 (1.4–3.7%)	183	239	198	103	39	762
	Quintile 3 (3.7–8.1%)	79	195	223	186	79	762
	Quintile 4 (8.1–17.9%)	33	112	182	267	171	765
	Quintile 5 Highest risk (17.9–91.1%)	2	27	75	191	476	771
	Total	777	777	760	771	771	3856

**Figure 1:**
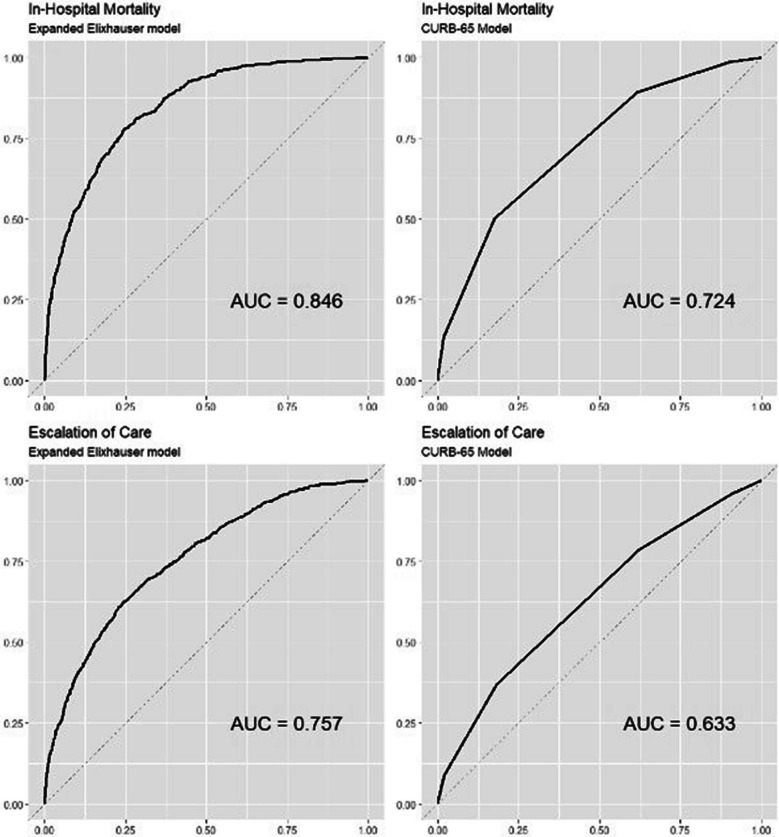
Receiver operating characteristic curves for the expanded Elixhauser model and CURB-65 score to predict the risk of death and the escalation of care among 3856 adult patients hospitalized for pneumonia.

Figure 1 shows that our case mix model predicted the study outcomes better than the CURB-65 score. Our case mix model achieved a c-statistic of 0.846 and 0.757 to predict in-hospital mortality and escalation of care, respectively. The CURB-65 score, in contrast, achieved c-statistic of 0.724 and 0.633, respectively. Our model also outperformed the CURB-65 with regard to predicting hospital length of stay, with an *R*^2^ of 0.285 vs. 0.057, respectively.

Table 3 gives us a sense of how our case mix model was able to achieve better prediction than the CURB-65 score—namely, through increased discrimination. Table 3 shows the risk of death based on the expanded Elixhauser model compared to the risk of death based on the CURB-65 score for our study population. This table shows that our risk score has much better discrimination than the CURB-65 model. For patients identified as very low risk or very high risk by the CURB-65 score, our score overwhelmingly agrees. However, for the vast majority of patients with a CURB-65 score of 1, 2, or 3, our score provides a wide range of predicted risk for in-hospital death. This implies that patients who “look” very similar to the CURB-65 score “look” quite different when examined with our case mix model, which could enable better decisions about whom to admit or send home. We also produced a related figure (Online Appendix A) which shows how our score correctly identifies the great majority of patients who experienced either study outcome (in-hospital mortality or escalation of care) within each CURB-65 score stratum as being higher-risk patients.

**Table 3:** Quintiles of Predicted Risk of In-Hospital Mortality Based on the Expanded Elixhauser Model Compared with CURB-65 Score, Among 3856 Adult Patients Hospitalized for Pneumonia

		CURB-65 score						
		0 Lowest risk	1	2	3	4	5 Highest risk	Total
Risk as predicted by the expanded Elixhauser model	Quintile 1 Lowest risk (0.3–1.4%)	231	353	180	32	0	0	796
	Quintile 2 (1.4–3.7%)	57	288	342	74	1	0	762
	Quintile 3 (3.7–8.1%)	24	190	432	113	3	0	762
	Quintile 4 (8.1–17.9%)	13	135	418	178	21	0	765
	Quintile 5 Highest risk (17.9–91.1%)	4	55	309	305	89	9	771
	Total	329	1021	1681	702	114	9	3856

Table 4 shows the results of the Hosmer–Lemeshow analysis for the five risk strata of our expanded Elixhauser model, from lowest risk (Quintile 1) to highest risk (Quintile 5). We also show the results of the Hosmer–Lemeshow analysis for the CURB-65 score, from lowest risk (score = 0) to highest risk (score = 5). Overall, model calibration was good for both models.

**Table 4:** Hosmer–Lemeshow Test of Calibration for the Expanded Elixhauser Model to Predict (a) Mortality and (b) Escalation of Care Among 3856 Adult Patients Hospitalized for Pneumonia

Risk group	Group size	Observed	Expected
*a*			
1	386	1 (0.26%)	1.78 (0.46%)
2	386	4 (1.04%)	3.25 (0.84%)
3	386	4 (1.04%)	5.41 (1.40%)
4	386	7 (1.81%)	9.07 (2.35%)
5	386	14 (3.63%)	14.65 (3.80%)
6	386	31 (8.0%)	23.31 (6.0%)
7	386	40 (10.4%)	37.02 (9.6%)
8	386	52 (13.5%)	58.20 (15.1%)
9	386	99 (25.6%)	95.08 (24.6%)
10	386	203 (53.1%)	207.23 (54.2%)
*b*			
1	386	9 (2.30%)	9.94 (2.60%)
2	386	13 (3.40%)	16.81 (4.40%)
3	386	20 (5.2%)	23.42 (6.1%)
4	386	46 (11.9%)	31.54 (8.2%)
5	386	37 (9.6%)	40.88 (10.6%)
6	386	51 (13.2%)	52.61 (13.6%)
7	386	66 (7.1%)	68.62 (17.8%)
8	386	96 (24.9%)	90.44 (23.4%)
10	386	126 (32.6%)	127.28 (33.0%)
11	386	218 (57.1%)	220.46 (57.7%)

## DISCUSSION

We examined a dataset of over 3856 admissions for pneumonia to the IM service of a large, tertiary-care hospital. Our expanded Elixhauser model achieved meaningfully superior prediction of in-hospital mortality and the need for escalation of care compared with the CURB-65 score, which is currently the one used at our hospital and at many others. For example, CURB-65 had a c-statistic for predicting mortality of 0.724, compared to 0.846 for our model, an extremely large difference in predictiveness. As a point of comparison, adding c-reactive protein to traditional cardiovascular risk factors did not measurably increase the ability to predict cardiovascular event risk (0.78 vs. 0.78).^13,14^ We also showed how our model was able to assign quite different levels of risk to patients who were assigned to the same risk group by the CURB-65 model (Table 3 and Fig. 1).

While the PSI could have provided somewhat more detail than the CURB-65 score, we did not compare it to PSI, because not all of our patients had all of the necessary data elements to calculate PSI—including information from an arterial blood gas (serum pH and pO_2_) and a chest X-ray (pleural effusion). A major advantage of our score is that it is calculable with information that was readily available on all of the patients in our sample, and does not rely on tests that are only performed on a minority of patients (arterial blood gas) or are not immediately available as structured data (X-ray). We compared it to the CURB-65 because it can also be calculated without X-ray or blood gas results. In addition, CURB-65 is the score currently in use at SZ, so it made sense to compare with it.

Of the two major existing scores (PSI and CURB-65), the PSI has been around longer. Fine et al. previously described results from the Pneumonia PORT cohort study on hospital admission decisions for patients with community-acquired pneumonia,^15^ prognosis of patients hospitalized with community-acquired pneumonia,^16^ validation of a pneumonia prognostic index, and analysis of hospital discharge decisions for patients with community-acquired pneumonia.^16^ Lim and colleagues subsequently developed CURB-65, a simple six-point score based on confusion, urea, respiratory rate, blood pressure, and age to stratify patients with pneumonia.^3^ Both models use demographic and clinical variables to predict 30-day mortality;^2^ however, the PSI identifies larger proportions of patients as low risk and has a higher discriminative power in predicting mortality.^4^ Unlike the PSI, whose utility as an aid to admission decisions has been proven with empirical research,^17^ there is less direct evidence that CURB-65 is effective in guiding the initial site of treatment. The recommendation of the American Thoracic Society (ATS) is to use the PSI as an adjunct to clinical judgment to guide the initial site of treatment.^18^

Risk stratification scores are developed in multiple stages that include deriving the score, validating the score, and finally, implementing it and evaluating its impact.^19^ In the first step, the risk stratification score is derived and validated internally, often using split-sample validation, and often retrospectively. This is followed by external retrospective validation, i.e., in a different dataset, such as from an alternate clinical site. Finally, the score should be deployed or implemented prospectively to guide care, which can demonstrate that its use changes clinical outcomes in a significant way compared to the previous system (which may be clinical gestalt).^19^

The derivation of our expanded Elixhauser model accomplishes only the first step in this multi-step process. However, our preliminary results are very promising. Our score, which is a general-purpose score for any admission to an IM service, performed significantly better than the CURB-65 score, which is in common use and specifically made for pneumonia. Our score is practical in that it is comprised of data that is immediately available at the time of admission. It is evident in Table 3 as to why our score outperforms the CURB-65. For example, patients assigned a uniform, medium risk by the CURB-65 score are assigned a much broader range of risks by our score.

While our results are promising, our study does have limitations. As explained above, truly validating such a risk prediction score is a laborious multi-step process, and the next steps for our score would be external validation, followed by prospective evaluation of clinical utility. Another weakness of our score is that it is based on single-center data. Finally, any risk score is subject to clinical judgment, and there are reasons to hospitalize patients classified as low-risk, such as patients who live alone or are unable to take oral medication. Because we did not manually review the charts, we would not know which patients would fit this description.

In summary, we found that our risk prediction score, which can be calculated using data which are available in most modern hospitals, clearly outperformed the CURB-65 score in predicting in-hospital mortality and escalation of care for patients with pneumonia. Important next steps for our risk prediction score would be external validation, followed by prospective evaluation of its clinical utility. Ultimately, our expanded Elixhauser model could be deployed efficiently to identify and appropriately triage patients with pneumonia to a standard hospital bed, an intensive care bed, home, or even a home hospitalization setting.

## Supplementary Information

Below is the link to the electronic supplementary material.

ESM 1 (DOCX 171 KB)

## Data Availability

We will share our statistical code and study protocol upon request. SZMC data are available upon reasonable request and after completing a data use agreement.

## Abbreviations

ATS: American Thoracic Society
BiPAP: Bi-level positive pressure ventilation
Covid-19: Coronavirus disease 2019
CURB-65: Confusion, urea, respiratory rate, blood pressure, age ≥ 65
IM: Internal medicine
PORT: Pneumonia Patient Outcomes Research Team
PSI: Pneumonia Severity Index
SZMC: Shaare Zedek Medical Center
