# Supplementary figures and images for: Improving Discrimination in Predicting Level of Care Needed for Patients Admitted with Pneumonia
Source: J Gen Intern Med. 2025 May 22;40(13):3051–7. doi: 10.1007/s11606-025-09610-7 (PMC12508420; doi:10.1007/s11606-025-09610-7)

Appendix A.


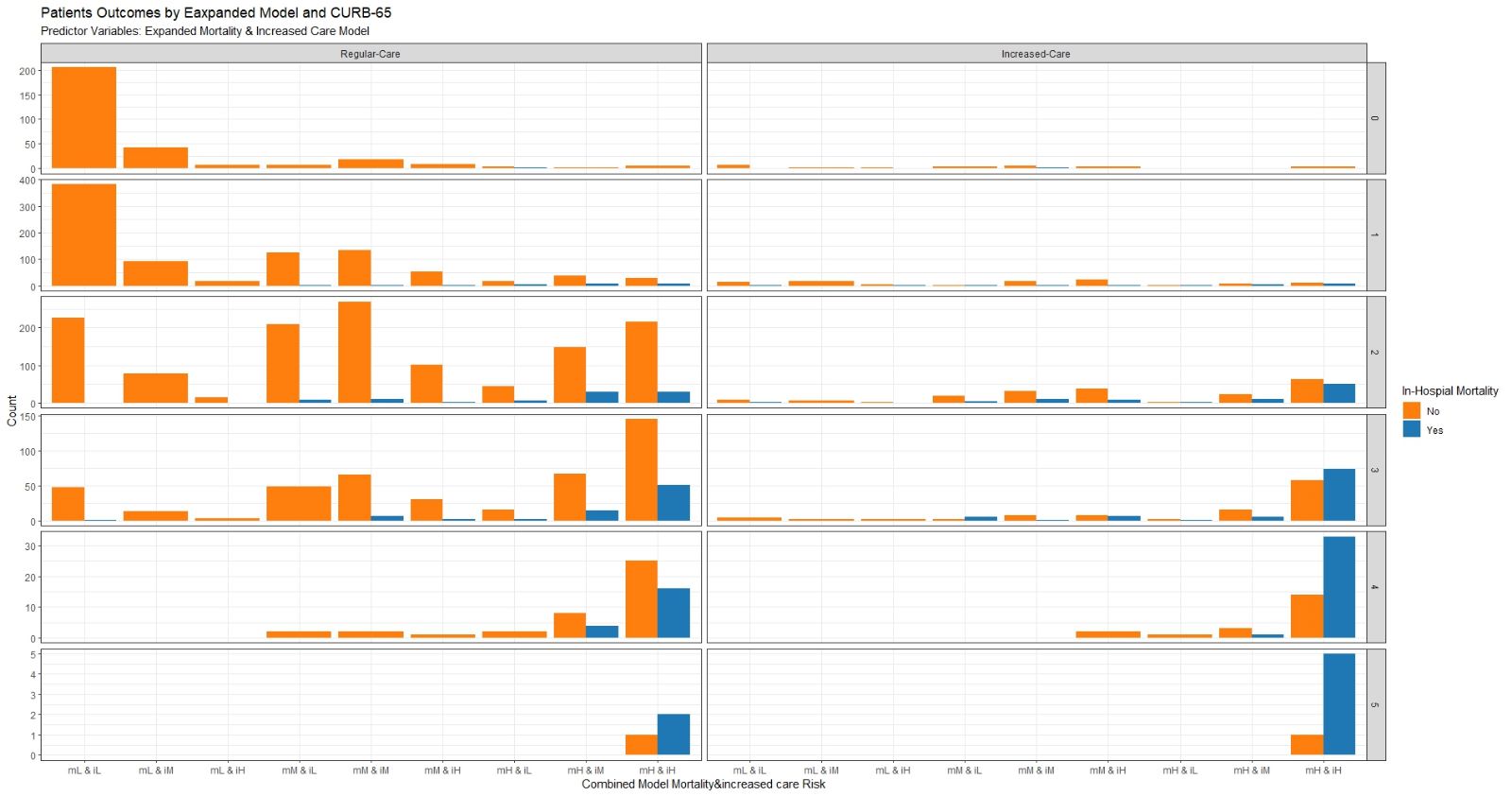

Supplement: Supplementary file 1 — (DOCX 171 KB) [file 11606_2025_9610_MOESM1_ESM.docx]
